# *Haemanthus coccineus* extract and its main bioactive component narciclasine display profound anti-inflammatory activities *in vitro* and *in vivo*

**DOI:** 10.1111/jcmm.12493

**Published:** 2015-03-05

**Authors:** Simone Fuchs, Louise T Hsieh, Werner Saarberg, Clemens A J Erdelmeier, Thomas A Wichelhaus, Liliana Schaefer, Egon Koch, Robert Fürst

**Affiliations:** a Institute of Pharmaceutical Biology, Biocenter, Goethe-UniversityFrankfurt/Main, Germany; b Department of Pharmacy, Pharmaceutical Biology, University of MunichMunich, Germany; c Institute of Pharmacology and Toxicology/ZAFES, Medical School, Goethe-UniversityFrankfurt/Main, Germany; d Preclinical Research, Dr. Willmar Schwabe GmbH & Co. KGKarlsruhe, Germany; e Institute of Medical Microbiology and Infection Control, Medical School, Goethe-UniversityFrankfurt/Main, Germany

**Keywords:** adhesion molecules, endothelium, *Haemanthus coccineus* extract, inflammation, isocarbostyril alkaloid, narciclasine, leucocyte-endothelial cell interactions, NFκB

## Abstract

*Haemanthus coccineus* extracts (HCE) have traditionally been used to treat a variety of diseases, like febrile colds or asthma. Since new therapeutic options against inflammatory processes are still urgently needed, we aimed to pharmacologically characterise the anti-inflammatory potential of HCE*in vitro* and *in vivo* and to identify the underlying bioactive component(s). The action of HCE on oedema formation and leucocyte infiltration were analysed in two murine models of inflammation (dermal oedema induced by arachidonic acid and croton oil; kidney injury caused by unilateral ureteral obstruction). The interaction of leucocytes with endothelial cells (ECs) as well as the activation parameters of these two cell types were analysed. Moreover, the nuclear factor κB (NFκB) pathway was investigated in detail in ECs. Using different fractions of HCE, the bioactive principle was identified. *In vivo*, HCE (450 mg/kg orally or 2 mg/kg intraperitoneally) inhibited oedema formation, leucocyte infiltration and cytokine synthesis. *In vitro*, HCE (100–300 ng/ml) blocked leucocyte-EC interaction as well as the activation of isolated leucocytes (cytokine synthesis and proliferation) and of primary ECs (adhesion molecule expression). HCE suppressed NFκB-dependent gene transcription in the endothelium, but did not interfere with the NFκB activation cascade (IκB degradation, p65 nuclear translocation and NFκB DNA-binding activity). The alkaloid narciclasine was elucidated as the bioactive compound responsible for the anti-inflammatory action of HCE. Our study highlights HCE and its main alkaloid narciclasine as novel interesting approach for the treatment of inflammation-related disorders.

## Introduction

Activation of inflammatory processes is a physiologically protective host response to tissue damage, infections or alterations caused by malignant cells. However, in many chronic diseases, like atherosclerosis, rheumatoid arthritis, asthma or chronic kidney disease, it contributes to pathological conditions 1–3. For most of these inflammation-related disorders, the conventional treatment including non-steroidal anti-inflammatory therapeutics, glucocorticoids or immunosuppressives is not always effective and often causes severe side effects. Therefore, new anti-inflammatory strategies are desperately needed to improve the benefit-to-risk ratio of the treatment 4. Initial steps of inflammation involve cytokine-mediated activation of leucocytes 5 and of the vascular endothelium 6. In response to pro-inflammatory mediators, such as tumour necrosis factor alpha (TNFα), the vascular endothelium strongly increases its expression of the cell adhesion molecules intercellular cell adhesion molecule-1 (ICAM-1), vascular cell adhesion molecule-1 (VCAM-1) and endothelial leucocyte adhesion molecule-1 (E-selectin) 7,8, a crucial step for the extravasation of leucocytes into the inflamed tissue 9,10. The interaction between leucocytes and the vascular endothelium has been recognised as an attractive target for the therapy of numerous disorders and diseases including excessive inflammatory responses 11.

Plant-derived natural products are still a very important source of therapeutically effective agents 12–14. Natural compound structures provide enormous chemical diversity and highly specific biochemical activity that make them valuable lead structures 15. *Haemanthus coccineus* extracts (HCE), especially preparations of the bulbs, have been used in the traditional South African medicine as a diuretic agent or for the treatment of inflammation-associated conditions including febrile colds, asthma, ulcers or wounds 16–18. Plants of the Amaryllidaceae family are well-known for their variety of pharmacologically active alkaloids 19. Narciclasine (Narc), an isocarbostyril alkaloid found in the bulbs of *H. coccineus*, has previously been shown to induce apoptosis in different tumour cell lines and to be highly selective for cancer cells compared to non-cancerous cells 20,21.

In this study, we have suggested that HCE exerts profound anti-inflammatory actions *in vitro* and *in vivo* by influencing endothelial and immune cells leading to the attenuation of cytokine expression and leucocyte extravasation. Moreover, we aimed to determine the bioactive principle of the extract responsible for these effects.

## Materials and methods

### Compounds

*Haemanthus coccineus* extracts, extract fractions and Narc were kindly provided by Dr. Willmar Schwabe GmbH & Co. KG (Karlsruhe, Germany). The extract was prepared from dried bulbs of *H. coccineus* with 60% ethanol (w/w). Drug extract ratio was 50:1. The resulting HCE was adjusted to a content of 2.2% Narc. The HCE was partitioned between ethyl acetate and water. The dried ethyl acetate phase was subsequently chromatographed through Sephadex LH-20 using 100% methanol as eluent. From this separation, a crude Narc fraction was obtained. Crystallisation of this fraction from methanol–acetone 1:1 lead to pure Narc. The structure of the isolate was identified by MS and NMR. The remaining fractions (Narc-free) from Sephadex LH-20 separation were further separated with reversed-phase HPLC to give complex basic alkaloid fractions (Dragendorff-positive reaction, thin-layer chromatography) that could not be further fractionated. *Haemanthus coccineus* extracts, its fractions and Narc were solubilised in DMSO (Sigma-Aldrich, Taufkirchen, Germany). Stock solutions at 30 mg/ml (HCE), 10 mg/ml (extract fractions) and 3 mM (Narc) were stored at −20°C. Substances were diluted in growth medium (concentrations as described in the respective passages) without exceeding a final DMSO concentration of 0.1%. Recombinant human TNFα was from PeproTech (Hamburg, Germany), formaldehyde (16%, methanol-free, ultra pure) was from Polysciences (Eppelheim, Germany). Heparin, 2-mercaptoethanol, croton oil, arachidonic acid (AA), dexamethasone (DEX), Tween® 80, concanavalin A (Con A), lipopolysaccharide (LPS) and fluorescein isothiocyanate (FITC)-dextran (40 kD) were purchased from Sigma-Aldrich. [^3^H]-thymidine was from GE Healthcare (Freiburg, Germany). PEG 400, agar and acetone were obtained from Merck (Darmstadt, Germany). Tepoxalin (TEP, Zubrin® 100 mg) was purchased from Essex-Tierarznei (Munich, Germany) and Isofluran CP® was obtained from CP-Pharma (Burgdorf, Germany).

### Animals

All animal care and experimental procedures were in accordance with the guidelines of the German Animal Protection Law and were approved by the Ethics Review Committee for Laboratory Animals of the District Government of Darmstadt (Germany) and Karlsruhe (Germany), respectively. C57BL/6 mice were purchased from Charles River Laboratories (Sulzfeld, Germany). Naval Medical Research Institute (NMRI) mice were provided by Janvier Labs (Le Genest-Saint-Isle, France).

### Cell culture

Primary human umbilical vein endothelial cells (HUVECs) were purchased from PELOBiotech (Martinsried, Germany). The human microvascular endothelial cell line CDC/EU.HMEC-1 22 was kindly provided by the Centres for Disease Control and Prevention (CDC, Atlanta, GA, USA) and was used until passage 30. HMECs were exclusively used in the THP-1 adhesion and permeability assay. Endothelial cells (ECs) were cultured in EC growth medium (PELOBiotech), which was supplemented with 10% heat-inactivated foetal bovine serum (FBS, Biochrom, Berlin, Germany), 100 U/ml penicillin (PAN-Biotech, Aidenbach, Germany), 100 μg/ml streptomycin (PAN-Biotech) and 0.25 μg/ml amphotericin B (PAN-Biotech) under constant humidity at 37°C in an atmosphere containing 5% CO_2_. Human neutrophil granulocytes were purified from peripheral blood of healthy volunteers using CD15 MicroBeads (Miltenyi, Bergisch Gladbach, Germany). The monocyte-like cell line THP-1 (ACC-16) was kindly provided by the Leibniz Institute for German Collection of Microorganisms and Cell Cultures (DSMZ, Braunschweig, Germany) and cultured as described by Fischer *et al*. 23. Lymphocytes were isolated from spleens of male C57BL/6 mice by gentle disruption and homogenization of the spleens and final density gradient centrifugation with Lymphodex (Fresenius, Bad Homburg, Germany). Lymphocytes were washed two times in Hank's Balanced Salt Solution (HBSS) and were cultured in complete RPMI-1640 medium (Sigma-Aldrich) supplemented with 10% heat-inactivated FBS (Sigma-Aldrich), 2 mM glutamine (Sigma-Aldrich), 100 U/ml penicillin, 100 μg/ml streptomycin, 0.25 μg/ml amphotericin B (antibiotic/antimycotic solution, Sigma-Aldrich) and 50 μM 2-mercaptoethanol under constant humidity at 37°C in an atmosphere containing 5% CO_2_. Mouse peritoneal macrophages were gained by injecting male NMRI mice intraperitoneally with 45 mg thioglycollate (Sigma-Aldrich) in 1.5 ml sterile H_2_O. Four days later, cells were recovered from 12 animals by peritoneal lavage with 5 ml HBSS containing heparin (0.04%). Activated macrophages were collected by centrifugation, washed two times with HBSS and cultured under the same conditions as murine lymphocytes.

### Dermal ear oedema model

A local inflammation in male NMRI mice was induced by the epicutaneous application of croton oil (2.5 μg/μl) or AA (82 nM), each in acetone, to the right ear. To the left ear, only acetone was applied. *Haemanthus coccineus* extracts was administered orally at doses ranging from 50 to 450 mg/kg 1 hr before croton oil or AA was applied. After six (croton oil) or after one (AA) hour, the animals were killed. Tissue plugs were punched out uniformly from each ear and plug weight was determined. The reduction of oedema formation was calculated as [1 − (*W*_t_/*W*_c_) × 100], with *W*_t_ and *W*_c_ representing the weight difference between plugs from the left and the right ear in treated or control mice. Dexamethasone-treated (0.3 mg/kg) or tepoxalin-treated (100 mg/kg) mice were used as positive control group. For visualising the presence of granulocytes in the tissue, mice ear paraffin sections (4 μm) were stained with a naphthol-AS-D-chloroacetate-esterase kit (Sigma-Aldrich) in accordance with the manufacturer's protocol. The intensity of staining was evaluated microscopically per high power field (HPF, 400×).

### Unilateral ureteral obstruction model

Obstruction of the left ureter was performed in 2-month-old male C57BL/6 mice as reported previously 24. Sham-operated mice served as control groups. Mice were divided into four groups: sham-operated animals receiving vehicle (PBS), sham-operated mice treated with HCE, unilateral ureteral obstruction (UUO) mice receiving PBS and UUO mice treated with HCE. Vehicle or HCE (2.0 mg/kg) was intraperitoneally administered to mice daily. Kidneys and plasma were analysed at day three after ligation of the left ureter.

### Renal immunohistochemistry

Serial sections (4 μm) of paraffin-embedded samples were processed for immunohistochemical studies using rat anti-mouse F4/80 antiserum (macrophage marker; AbD Serotec, Puchheim, Germany) and horseradish peroxidase/3,3′-diaminobenzidine techniques 25,26. Incubation with the primary antibody was performed overnight at 4°C. Counterstaining was performed with Meyer's Haematoxylin (Sigma-Aldrich). The specificity of immunostaining was confirmed by omitting the primary antibody and by non-immune serum/unspecific IgG. Infiltrating macrophages were counted in 15 randomly selected non-overlapping HPFs (400×) of renal sections and calculated per field using Soft Imaging System (Olympus, Hamburg, Germany).

### Quantitative polymerase chain reaction

Total RNA from mouse kidneys was isolated using TRI reagent (Sigma-Aldrich) as described previously 25 and reversely transcribed using the Verso cDNA synthesis Kit (Thermo Fisher Scientific, Schwerte, Germany). cDNA was amplified using TaqMan Gene Expression assays. Probes for mouse *Tnf* (Mm_00443260_g1), chemokine (C-C motif) ligand (*Ccl2*, Mm00441242_m1) and glyceraldehyde-3-phosphate dehydrogenase (*Gapdh*, Mm03302249_g1) were purchased from Life Technologies (Darmstadt, Germany). Amplification and detection were performed with an ABI prism 7500 Sequence Detection System (Life Technologies). The threshold cycle for the gene of interest was normalized to that of *Gapdh*.

### CCL2 plasma protein level

Plasma samples were used to determine CCL2 protein levels by mouse-specific ELISA (R&D Systems, Wiesbaden, Germany) in accordance to the manufacturer's protocol in duplicates.

### Synthesis of pro-inflammatory cytokines in murine peritoneal exudate cells

Mouse peritoneal activated macrophages were gained as described in the section cell culture. About, 4 × 10^5^ cells per well were cultured in 96-well plates (TPP, Trasadingen, Switzerland). Cytokine release was assayed by an ELISA as described previously 27. Briefly, cells were pre-treated with HCE for 30 min., and then cytokine production was stimulated by the addition of LPS from *Escherichia coli* (1 μg/ml) for 24 hrs. After cell lysis, the amount of synthesised pro-inflammatory cytokines was quantified in supernatants with commercially available ELISA kits for TNFα (R&D Systems), interleukin (IL)-6 (R&D Systems) and IL-β (R&D Systems) in accordance to the manufacturer's protocol.

### Lymphocyte proliferation

Lymphocytes were gathered from murine spleens as described in the section cell culture. Proliferation assays with murine lymphocytes were performed as described previously 27. Briefly, lymphocytes (10^5^ cells per well) were cultured in 96-well plates (Greiner Bio-One, Frickenhausen, Germany) and treated as described. As mitogens Con A or LPS from *Salmonella typhosa* were used at final concentration of 2.5 μg/ml. Cells were pulse-labelled with [^3^H]-thymidine (0.5 μC/25 μl per well) during the final 6 hrs of the 72 hrs incubation period and then harvested onto fibre glass filters type G-10 (Berthold Detection Systems, Pforzheim, Germany) using the semiautomatic cell counter H 110 (Berthold Detection Systems). Incorporation of radioactively labelled thymidine was determined directly by the position-sensitive proportional counter LB284RA (Berthold Detection Systems).

### THP-1 adhesion assay

HMECs were cultured to confluence in 24-well plates (VWR, Darmstadt, Germany) and exposed to HCE as described. Cell-Tracker™ Green-labelled (10 μM; Life Technologies) THP-1 cells were allowed to adhere on a confluent HMEC monolayer for 1 hr. Non-adherent THP-1 cells were removed by washing three times with pre-warmed PBS (containing Ca^2+^ and Mg^2+^). After lysis, fluorescence was analysed at 535 nm using a Varioskan® Flash plate reader (Thermo Fisher Scientific, Langenselbold, Germany).

### Neutrophil granulocyte adhesion assay

Neutrophil granulocytes were added to confluent HUVECs that were pre-treated as indicated and allowed to adhere for 30 min. After lysis, myeloperoxidase activity, an enzyme solely expressed in neutrophils, was measured in the supernatants as described previously 28.

### Flow cytometric analysis

Human umbilical vein ECs were trypsinized, formalin-fixed (4%, methanol-free), washed and incubated with FITC-labelled anti-CD54 (ICAM-1) antibody (BIOZOL, Eching, Germany), FITC-labelled anti-CD106 (VCAM-1) antibody (Becton Dickinson, Heidelberg, Germany) or PE-labelled anti-CD62E (E-selectin) antibody (Becton Dickinson). Stained HUVECs were analysed by flow cytometry (FACSCanto II or FACSVerse, Becton Dickinson) 28. Apoptotic cell rates were quantified by measuring the subdiploid DNA content and analysed by flow cytometry (FACSCanto II) according to the method of Nicoletti *et al*. 29.

### Dual luciferase nuclear factor κB reporter gene assay

The assay was performed as described previously 28. Briefly, HUVECs (70–80% confluent) were transiently cotransfected with the firefly luciferase reporter vector pGL4.32[luc2P/nuclear factor (NF)κB-RE/Hygro] (Promega Corp., Heidelberg, Germany) and the *Renilla* luciferase control reporter vector (determination of transfection efficiency) pGL4.74[hRluc/TK] (Promega) at a ratio of 10:1 using the Targefect-HUVEC kit (Targetingsystems, El Cajon, CA, USA) and cultured for 18 hrs. Then, cells were treated as described, lysed and the NFκB promoter activity was measured by the Dual-Luciferase® Reporter Gene assay (Promega) according to the manufacturer's instructions. Luciferase activity was detected using a Berthold Orion II Luminometer (Berthold Detection Systems).

### Western blot analysis

Human umbilical vein EC cell lysates were prepared as reported previously 28. Proteins were separated by SDS-PAGE and transferred to polyvinylidene difluoride membranes (Bio-Rad Laboratories, Munich, Germany) by tank electroblotting. Membranes were blocked with bovine serum albumin (BSA, Sigma-Aldrich). The following primary antibodies were used: mouse monoclonal β-tubulin (1:1000; Santa Cruz Biotechnology, Heidelberg, Germany), rabbit polyclonal anti-IκBα (1:1000; Santa Cruz Biotechnology), rabbit polyclonal anti-phospho (T180/Y182)-p38 (1:1000; Cell Signaling/New England Biolabs, Frankfurt am Main, Germany), rabbit polyclonal anti-p38 (1:1000; Cell Signaling/New England Biolabs), mouse monoclonal anti-phospho (T202/Y204)-ERK1/2 (1:1000; Cell Signaling/New England Biolabs), rabbit polyclonal anti-ERK1/2 (1:1000; Cell Signaling/New England Biolabs), rabbit monoclonal anti-phospho (Y705)-STAT3 (1:1000; Cell Signaling/New England Biolabs) and mouse monoclonal anti-STAT3 (1:1000; Cell Signaling/New England Biolabs). For detection, mouse monoclonal anti-β-actin (1:25,000; Sigma-Aldrich), goat-anti-mouse (1:2000; Santa Cruz Biotechnology), and goat-anti-rabbit (1:2000; Santa Cruz Biotechnology) antibody labelled with horseradish-peroxidase were used.

### NFκB p65 translocation

Human umbilical vein ECs were cultured until confluence on collagen-coated μ-slides (Ibidi, Martinsried, Germany) and treated as indicated. Cells were washed, fixed with Accustain™ (Sigma-Aldrich), permeabilized by 0.2% Triton X-100 (Merck), blocked in 0.2% BSA and incubated with an anti-NFκB p65 (Santa Cruz Biotechnology) primary and AlexaFluor 488-linked secondary antibody (Molecular Probes®/Life Technologies). Fluorescence microscopy was performed on a Zeiss Observer Z1 (Zeiss, Oberkochen, Germany). Quantification of p65 NFκB nuclear intensity was performed with ImageJ version 1.43u (National Institutes of Health, Bethesda, MD, USA).

### Electrophoretic mobility shift assay

Nuclear protein extracts were prepared from HUVECs, and electrophoretic mobility shift assay was performed as described previously 28.

### Cell viability assay

Human umbilical vein ECs were treated as indicated and metabolic activity was determined by the CellTiter-Blue® assay (Promega) as described previously 30. Viable cells retain the ability to reduce the non-fluorescent resazurin into resorufin, which is highly fluorescent. Fluorescence (ex: 560 nm; em: 590 nm) intensity was detected by a SPECTRAFluor Plus plate reader (Tecan, Crailsheim, Germany).

### Macromolecular permeability assay

HMECs were used to investigate macromolecular (FITC-dextran, 40 kD) endothelial permeability in a two compartment system (Transwell inserts; Corning, Wiesbaden, Germany). The assay was performed as reported previously 31.

### Broth microdilution susceptibility assay

Antimicrobial activity was evaluated by determining the minimum inhibitory concentration (MIC), *i.e*. the lowest concentration of HCE and Narc that prevents visible growth of a microorganism in a broth microdilution susceptibility test, following guidelines set by the Clinical and Laboratory Standards Institute (CLSI) 32. All MICs were determined in triplicates and quality control was performed according to CLSI recommendations.

### Statistical analysis

The numbers of independently performed experiments (*N*) are stated in the respective figure legends. In the case that nothing else is mentioned, bar graph data are expressed as means ± SEM. Statistical analyses were performed with the software Prism (version 5.04; GraphPad Software, San Diego, CA, USA). For evaluation of three or more groups one-way anova followed by Dunnett's significance correction test or Tukey's post-hoc test was used. *P* ≤ 0.05 were considered as statistically significant.

## Results

### HCE reduces oedema formation and neutrophil infiltration *in vivo*

**Figure 1:**
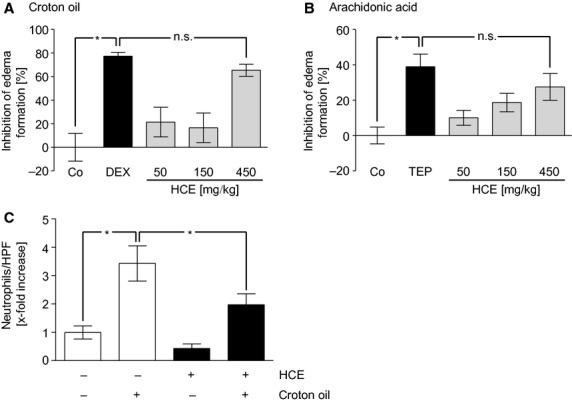
HCE diminishes oedema formation *in vivo*. The HC extract (50–450 mg/kg) was administered orally to mice 1 hr before croton oil (A) or arachidonic acid (B) was applied epicutaneously. After six (A) or after one (B) hour, the ear weight as a measure for oedema formation was determined. Dexamethasone (0.3 mg/kg; A) as well as tepoxalin (100 mg/kg; B) were used as positive control groups. *N* = 8, per group; *P < 0.05 *versus* control (Co); n.s. = not significantly different *versus* dexamethasone (DEX; A) or tepoxalin (TEP; B). (C) Quantification of neutrophil count per HPF. *N* (Co) = 22, *N* (HCE) = 30, *N* (Co + croton oil) = 28, *N* (HCE + croton oil) = 36; *P ≤ 0.05.

An *in vivo* model of an acute and local inflammation was used to investigate the anti-inflammatory potential of HCE. In this model, ear oedema was induced in mice by two different pro-inflammatory compounds: (*i*) In the croton oil-induced model, mice were treated orally with three different dosages of HCE (50, 150, 450 mg/kg) 1 hr before croton oil was epicutaneously applied. The highest dosage significantly decreased the ear weight differences (≈65%) between inflamed and non-inflamed ears, which clearly indicates a diminished oedema formation (Fig.1A). Notably, HCE was as potent as the glucocorticoid DEX. (*ii*) As a second pro-inflammatory agent, AA was used to evoke ear oedema formation. Oral pre-treatment with HCE (1 hr before AA was applied) dose-dependently attenuated the swelling of the AA-treated ear compared to the control ear (Fig.1B). Again, the anti-inflammatory potency of HCE (450 mg/kg) was comparable to that of an established non-steroidal anti-inflammatory drug (tepoxalin). Interestingly, no anti-inflammatory effect was observed when HCE (250 μg) was applied topically to the ear (data not shown). Indeed, the number of neutrophil granulocytes in the ear tissue was significantly reduced (≈50%) by HCE (450 mg/kg) in the croton oil model (Fig.1C). These data clearly hint towards a profound anti-inflammatory potential of HCE, which is associated with a reduced infiltration of neutrophils.

### HCE attenuates macrophage extravasation and CCL2 production after UUO

**Figure 2:**
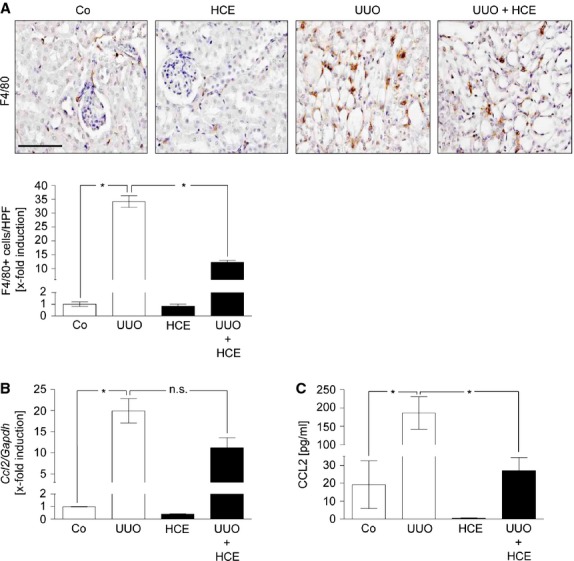
HCE exhibits anti-inflammatory activities in the UUO model. (A) Representative immunohistochemical staining of infiltrated macrophages (brown colour) in renal sections from HCE-treated mice after UUO (UUO+HCE), untreated mice (UUO), sham-operated mice (Co) or sham-operated mice by followed HCE treatment (HCE). Scale bar indicates 100 μm. Quantification of the infiltrated macrophages in the renal injured tissue was assessed per high power field. *N* (Co) = 15, *N* (UUO) = 14, *N* (HCE) = 15, *N* (UUO+HCE) = 15; *P ≤ 0.05. Determination of mRNA (B) or plasma protein levels (C) of renal CCL2 in total kidney homogenates. After UUO mice were treated with HCE (UUO + HCE) or left untreated (UUO). Sham-operated mice (Co) and sham-operated mice with subsequent HCE treatment (HCE) served as control groups. *N* (Co) = 3, *N* (HCE) = 3, *N* (UUO) = 3, *N* (UUO + HCE) = 5. *P ≤ 0.05; n.s. = not significantly different.

In a second *in vivo* model, which uses unilateral ureteral obstruction to trigger inflammatory events in murine kidneys, HCE (2 mg/kg/d for 3 days) was applied intraperitoneally. As shown in Figure2A, HCE blocked the UUO-induced infiltration of macrophages into tubulointerstitial regions of the obstructed kidney. Moreover, we analysed the levels of CCL2, an important chemoattractant for macrophages also known as monocyte chemotactic protein-1. *Haemanthus coccineus* extracts attenuated the synthesis of CCL2 mRNA in the kidney by 44% (Fig.2B) and inhibited the rise of this chemokine evoked by UUO in the blood by 86% (Fig.2C). Our results indicate that treatment with HCE strongly diminishes macrophage infiltration in the UUO model by decreasing CCL2 levels.

### HCE strongly blocks the release of pro-inflammatory cytokines and the proliferation of leucocytes *in vitro*

**Figure 3:**
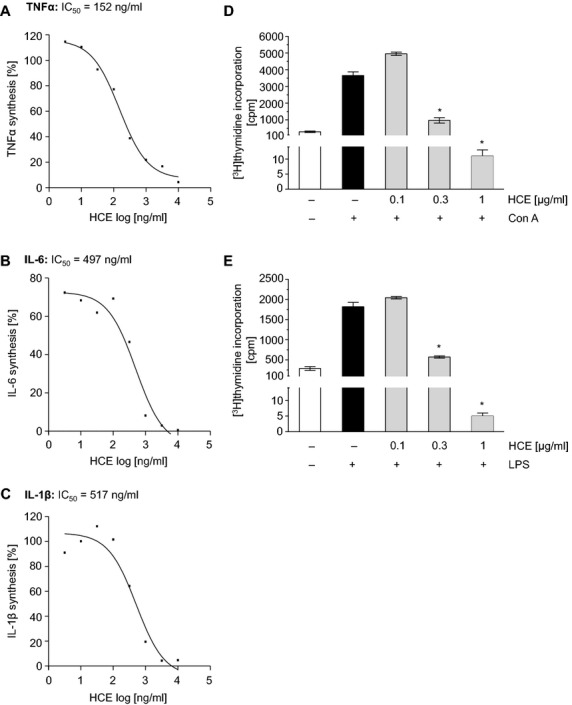
HCE inhibits pro-inflammatory cytokine synthesis and suppresses lymphocyte proliferation *in vitro*. Murine macrophages were pre-incubated with HCE (3 ng/ml–10 μg/ml) for 30 min. and then activated with LPS (1 μg/ml) for 24 hrs. The concentrations of TNFα (A), IL-6 (B) and IL-1β (C) in the cell culture were analysed by ELISA. *N* = 3. (D and E) Lymphocytes isolated from mice spleens were treated with HCE (100 ng/ml–1 μg/ml) and activated with Con A (2.5 μg/ml; D) or LPS (2.5 μg/ml; E) for 72 hrs. Subsequently, cells were pulse-labelled with [methyl-^3^H]thymidine (0.5 μC/25 μl per well) for 6 hrs. Thymidine uptake was measured and lymphocyte proliferation was evaluated in counts per minute (cpm). *N* = 4; *P ≤ 0.05 *versus* control.

We investigated whether HCE can interfere with the activation of macrophages and/or lymphocytes. LPS-activated macrophages from the murine peritoneum were treated *in vitro* with HCE. The extract concentration-dependently suppressed the secretion of the pro-inflammatory cytokines TNFα (Fig.3A), IL-6 (Fig.3B), and IL-1β (Fig.3C). The IC_50_ values were calculated as 152, 497, and 517 ng/ml, respectively. Interestingly, we could also observe a slight decrease of TNFα secreted by macrophages during renal injury (data not shown). Besides macrophages, HCE also affects isolated murine lymphocytes. Both the proliferation of T cells, induced by Con A (Fig.3D), and the proliferation of B cells, caused by LPS (Fig.3E), were strongly attenuated by HCE in a concentration-dependent manner. These data suggest that HCE can block the inflammation-associated activation of macrophages and lymphocytes.

### HCE reduces the adhesion of leucocytes to the endothelium and the expression of EC adhesion molecules

**Figure 4:**
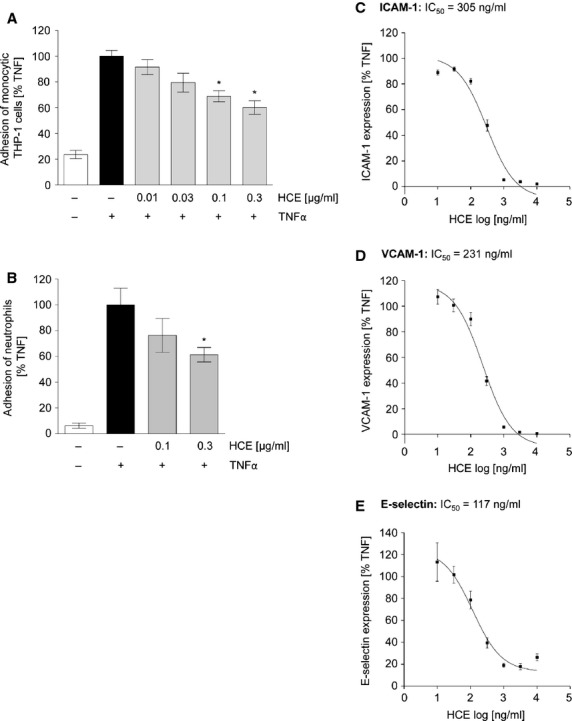
HCE strongly reduces leucocyte adhesion by blocking the expression of endothelial adhesion molecules. (A) After HCE pre-treatment (10–300 ng/ml for 30 min.) HMECs were treated with TNFα (10 ng/ml) for 6 hrs. Cell-Tracker™ Green-labelled THP-1 monocytic cells were added (3 × 10^5^ cells per well) and were allowed to adhere for 1 hr. The fluorescence of adhered monocytic THP-1 cells was measured (ex: 485 nm; em: 535 nm). *N* = 4; *P ≤ 0.05, *versus*TNFα. (B) Confluent primary endothelial cells were left untreated or were pre-incubated with HCE (0.1 and 0.3 μg/ml) for 30 min. Then they were treated with TNFα (10 ng/ml) for 24 hrs. Human neutrophil granulocytes were added (10^5^ cells per well) and were allowed to adhere for 45 min. To determine the amount of adhered neutrophils the activity of myeloperoxidase was measured. *N* = 3; *P ≤ 0.05 *versus*TNFα. (C and D) Adhesion molecule expression on the endothelial cell surface was determined by flow cytometry. HUVECs were either left untreated or were pre-incubated with HCE (10 ng/ml–10 μg/ml) for 30 min. Afterwards, cells were treated with TNFα (10 ng/ml) for 24 hrs (C, ICAM-1; D, VCAM-1) or for 6 hrs (E, E-selectin). *N* = 3.

Since leucocyte extravasation is tightly controlled by the endothelium, we investigated the influence of HCE on ECs. We found that the adhesion of monocyte-like THP-1 cells to TNFα-activated HMECs was concentration-dependently inhibited by HCE, which was exclusively applied to ECs (Fig.4A). Also the adhesion of freshly isolated human neutrophils onto a TNFα-activated monolayer of primary human ECs was clearly reduced by HCE (Fig.4B). Adhesion events are mediated by the up-regulation of EC adhesion molecules 33. Thus, we studied the action of HCE on the TNFα-triggered cell surface expression of ICAM-1 (Fig.4C), VCAM-1 (Fig.4D), and E-selectin (Fig.4E). *Haemanthus coccineus* extracts strongly suppressed the up-regulation of all three adhesion molecules in a clear concentration-dependent manner. IC_50_ values were calculated as 305 ng/ml for ICAM-1, 231 ng/ml for VCAM-1, and 117 ng/ml for E-selectin. Moreover, we proved that the applied concentrations of HCE had no cytotoxic effects on ECs. Neither the metabolic activity (Fig. S1A) nor the apoptotic cell rate (Fig. S1B) were affected at concentrations used in our experimental settings (10–300 ng/ml). Taken together, HCE strongly inhibits leucocyte adhesion to ECs by preventing the expression of EC adhesion molecules.

### HCE downregulates NFκB-dependent gene expression, but does not influence the activation cascade of endothelial NFκB

**Figure 5:**
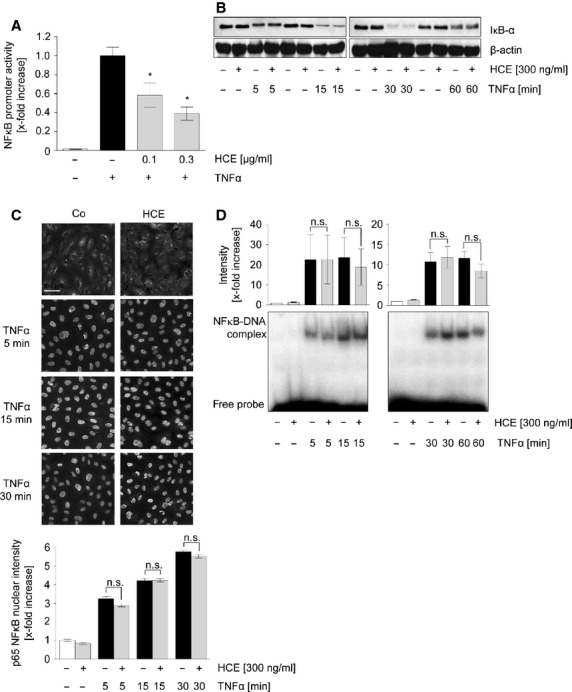
HCE suppresses TNFα-triggered NFκB promoter activity but does not affect canonical NFκB activation cascade. (A) Confluent HUVECs were cotransfected with an NFκB reporter vector (firefly luciferase) and a control vector (*Renilla* luciferase). 24 hours after transfection, cells were pre-treated with HCE (100 or 300 ng/ml) for 30 min. TNFα (10 ng/ml) was applied for 5.5 hrs. A Dual-Luciferase® Reporter Gene assay was used to analyse NFκB-dependent reporter gene expression, which is expressed as ratio of firefly luciferase/*Renilla* luciferase activity. *N* = 4; *P ≤ 0.05 *versus*TNFα. (B) Levels of IκBα and β-actin were assessed by Western blot analysis. One representative out of three independently performed experiments is shown, each. (C) The translocation of NFκB p65 subunit into the nucleus was visualized by immunocytochemistry and fluorescence microscopy. The median nuclear fluorescence intensity was analysed. *N* = 3; n.s. = not significantly different. (D) Nuclear extracts were prepared from HUVEC lysates and subsequently analysed for their NFκB DNA-binding activity *via* radioactive gel shift assay followed by densitometric analysis. *N* = 3; n.s. = not significantly different.

The expression of adhesion molecules is strongly regulated by the pro-inflammatory transcription factor NFκB. The impact of HCE on the NFκB-dependent promoter activity was investigated in ECs by a luciferase-based NFκB reporter gene assay. As shown in Figure5A, HCE concentration-dependently decreased NFκB promoter activity in TNFα-activated HUVECs. We have suggested that HCE might interfere with the activation cascade of NFκB. However, we could not detect any influence of HCE, neither on the TNFα-induced degradation of the NFκB inhibitor IκBα (Fig.5B), nor on the nuclear translocation of the NFκB subunit p65 (Fig.5C), or the DNA-binding activity of NFκB (Fig.5D). In summary, these findings provide evidence that HCE suppresses NFκB-dependent gene expression without interfering with the canonical activation cascade of this transcription factor.

### The anti-inflammatory effect of HCE can clearly be assigned to the isocarbostyril alkaloid Narc

**Figure 6:**
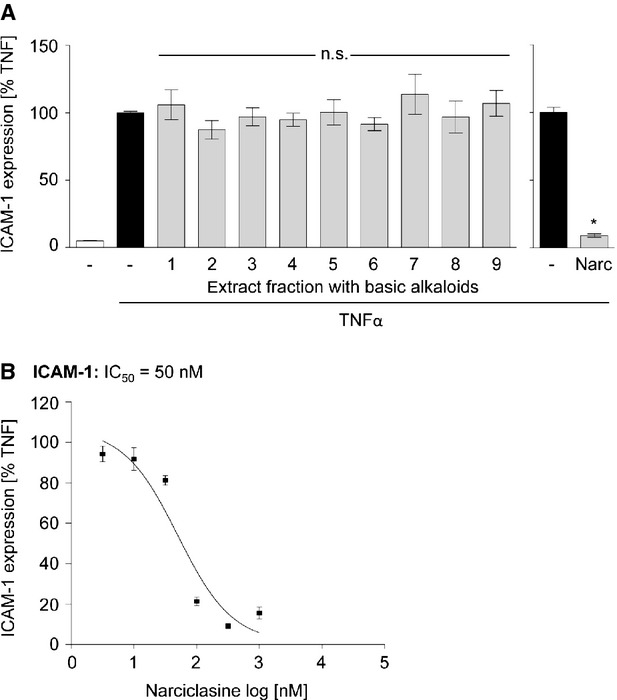
Narciclasine strongly inhibits the up-regulation of ICAM-1 on the endothelial surface, whereas extract fractions do not influence the adhesion molecule expression. (A and B) HUVECs were pre-treated with 300 ng/ml of extract fractions (A) or with several concentrations (3 nM–1 μM) of narciclasine (B) for 30 min. before TNFα-treatment (24 hrs). The levels of ICAM-1 on the cell surface were determined by flow cytometric analysis. *N* = 3; *P ≤ 0.05 *versus*TNFα, n.s. = not significantly different *versus*TNFα.

We aimed at identifying the bioactive compound(s) responsible for the anti-inflammatory action of HCE. Thus, we tested nine fractions obtained from the ethyl acetate extract of *H. coccineus* for their ability to affect the TNFα-induced ICAM-1 expression, which was used as robust read-out parameter. All subfractions, which contain (besides other compounds) the basic alkaloids of HCE, were applied at 300 ng/ml, but did not affect ICAM-1 expression (Fig.6A). In contrast, the isolated non-basic isocarbostyril alkaloid Narc, the main alkaloid of HCE, significantly reduced ICAM-1 expression with an IC_50_ value as low as 50 nM (Fig.6B). These findings highlight Narc as the major bioactive compound responsible for the anti-inflammatory action of HCE.

## Discussion and conclusion

Limited efficacy and intolerable side effects of the available anti-inflammatory agents, such as steroids, immunosuppressives, and non-steroidal anti-inflammatory drugs, have constantly motivated an intensive search for new compounds that inhibit the inflammatory response 4. Natural products have long been recognized as a key source for the development of novel therapeutic strategies to treat human disorders, including inflammatory diseases 34. In this study, we demonstrate for the first time that treatment with an extract from bulbs of *H. coccineus* and its major ingredient, the isocarbostyril alkaloid Narc, represent a novel and interesting anti-inflammatory approach.

Inflammatory events are characterized by changes in vascular permeability resulting in oedema formation. We clearly showed that HCE exhibits a pronounced anti-inflammatory and anti-oedematogenic activity *in vivo*, which was comparable to that of the glucocorticoid DEX or the COX/LOX inhibitor tepoxalin. Only the oral administration led to an anti-inflammatory action, the topical application of HCE was not effective (data not shown). Thus, the systemic availability of ingredients of HCE seem to be necessary for its *in vivo* activity against local ear oedema. Interestingly, HCE seems not to interfere with endothelial processes that regulate vascular barrier function, since macromolecular permeability was not altered by HCE (Fig. S2).

The most prominent action of HCE was the reduction in leucocyte infiltration both in the ear oedema and in the kidney injury model (UUO). This effect could in principle be evoked by an inhibition (*i*) of leucocyte and/or (*ii*) of EC activation: (*i*) Local expression of the chemokine CCL2 by immune or ECs of the injured kidney was suppressed by HCE on mRNA as well as on plasma protein level. Furthermore, HCE effectively inhibited the release of prominent pro-inflammatory cytokines (TNFα, IL-6, and IL-1β) from (isolated murine) macrophages and suppresses the proliferation of (isolated murine) lymphocytes. Thus, HCE is able to block the activation of different types of leucocytes. (*ii*) The up-regulation of EC adhesion molecules is a hallmark of inflammatory processes and crucial for leucocyte extravasation and tissue infiltration 35. *Haemanthus coccineus* extracts strongly inhibited the cell surface expression of all investigated adhesion molecules (ICAM-1, VCAM-1, and E-selectin). Interestingly, some disorders that have traditionally been treated with HCE in South Africa (*e.g*. asthma) are associated with inflammatory processes. Thus, we for the first time provide evidence for the rational basis of this usage, since we found that HCE effectively prevents both leucocyte and EC activation. Besides the anti-inflammatory action of HCE, we also investigated a potential antimicrobial effect of the extract. This might be of importance since many immunosuppressive therapies show infections as side effects 36,37. However, no antibacterial activity of HCE or Narc could be observed against relevant human pathogens at the given concentrations that reveal anti-inflammatory activity (Table S1).

Regarding the underlying mechanism, we demonstrated that HCE does not affect the pro-inflammatory signal transducers p38 MAPK, ERK1/2, and STAT3 (Fig. S3), but significantly inhibits NFκB-dependent gene transcription. The transcription factor NFκB is crucially involved in the pro-inflammatory activation of both leucocytes and ECs, since NFκB response elements are part of the promoter region of many cytokines and adhesion molecules 38. Surprisingly, none of the key players of the canonical NFκB activation cascade (IκB degradation, nuclear p65 translocation, NFκB DNA binding) were altered. Since NFκB DNA-binding can still occur, but NFκB-dependent gene expression is blocked, one could speculate that HCE could interfere with mRNA production, stability, or shuttling. This hypothesis is based on one of our former studies: We could show that flavopiridol, a potent inhibitor of cyclin-dependent kinases (CDKs), blocks NFκB promoter activity and the expression of NFκB-dependent genes. In parallel to our present findings, also flavopiridol did not influence any stage of the NFκB activation cascade. Instead, we found that flavopiridol was able to alter transcription elongation in the nucleus by inhibition of CDK9 39.

As a future perspective, it would be interesting to investigate whether HCE interferes with the death receptor (DR) pathway. Dumont *et al*. recently reported that Narc induces cell-specific apoptosis by inducing the initial caspases of DR pathways for the cell death surface receptor FAS and DR4 40. Besides tumour cells, also ECs express DR4, which binds to its ligand TNF-related apoptosis-inducing ligand. Activation of this receptor has been reported to participate in the regulation of EC activation including apoptosis, differentiation, inflammation, and proliferation 41.

By screening different fractions of HCE, we could show that basic alkaloids (*e.g*. coccine, haemanthamin, lycorine and montanine) are not active. We identified the non-basic alkaloid Narc to be responsible for the anti-inflammatory effect of HCE. Many studies report that Narc possesses profound anti-cancer activities *in vivo* and *in vitro* 21,42,43. Regarding inflammation, few reports have shown that Narc can protect against adjuvant-induced arthritis in mice 44–46. In accordance with our data, Yui *et al*. observed that Narc attenuates cytokine synthesis in murine macrophages. Interestingly, at least three direct targets of Narc have been suggested: In the 1970s, the alkaloid was discussed as inhibitor of protein synthesis by interfering with ribosomes of rabbit reticulocytes 47 or HeLa cells 48,49. Moreover, Narc can induce stress fibre formation in cancer cells by activating the small GTPase RhoA 50. In 2010, it was reported that Narc binds to and inhibits the transcription elongation factor eEF1A in melanoma cells 51. Leucocytes and ECs have as yet not been evaluated in this context. Nevertheless, regarding our results on the transcription factor NFκB (inhibition of gene expression without blocking NFκB activity), we could speculate that Narc might inhibit eEF1A also in ECs. However, we could not observe a global reduction in the protein synthesis resulting in cytotoxicity. Furthermore, from our permeability measurements, we do not have hints that Narc influences the EC shape and, thus, the cytoskeleton. In contrast to all these targets, we currently investigate the hypothesis that the alkaloid interferes with endothelial ion currents.

In summary, the results of our study provide a solid scientific explanation for the traditional use of extracts of *H. coccineus* as herbal remedy for the treatment of inflammation-related diseases. Moreover, we showed that HCE interferes with inflammatory processes in a dual manner: it affects both leucocytes and ECs. *Haemanthus coccineus* extracts inhibits leucocyte tissue infiltration by blocking the interaction of leucocytes with the endothelium, it attenuates leucocyte activation (proliferation as well as cytokine production), and decreases the up-regulation of adhesion molecules in the endothelium. These effects are based on the inhibition of NFκB-dependent gene expression. Taken together, our study suggests the use of HCE and its main alkaloid Narc as novel and promising approach to treat inflammatory conditions.

## Supporting Information

**Figure S1** Cell viability is affected by HCE only at very high concentrations. **Figure S2** HCE does not affect thrombin receptor-activating peptide (TRAP)-induced endothelial hyperpermeability. **Figure S3** HCE does not interfere with endothelial MAPK or STAT3 signalling pathway.

**Table S1** Minimum inhibitory concentration (MIC) of HCE and narciclasine against relevant human pathogens.
