# Supplementary figures and images for: Haemanthus coccineus extract and its main bioactive component narciclasine display profound anti-inflammatory activities in vitro and in vivo
Source: J Cell Mol Med. 2015 Mar 5;19(5):1021–32. doi: 10.1111/jcmm.12493 (PMC4420604; doi:10.1111/jcmm.12493)

Supporting information

Fig. S1

A

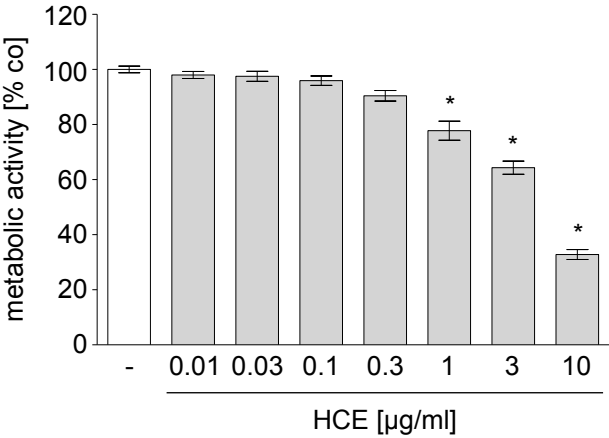

B

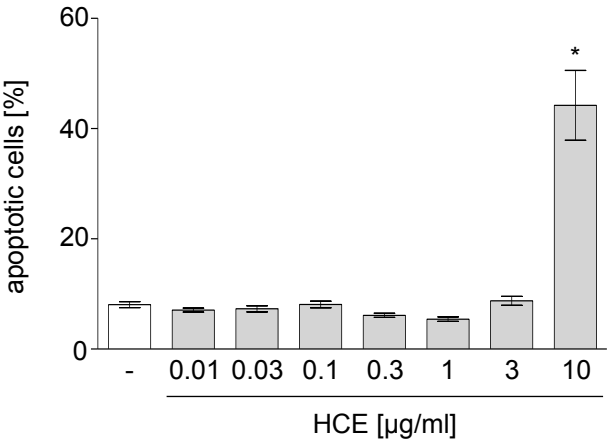

**Fig. S2**

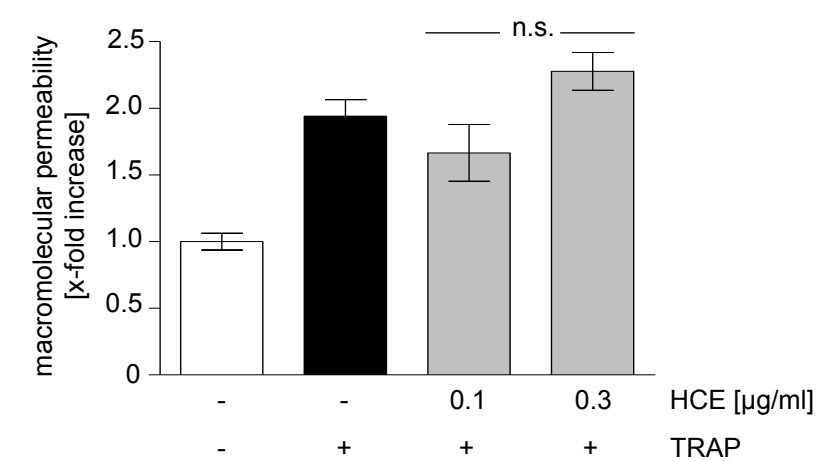

Fig. S3

A

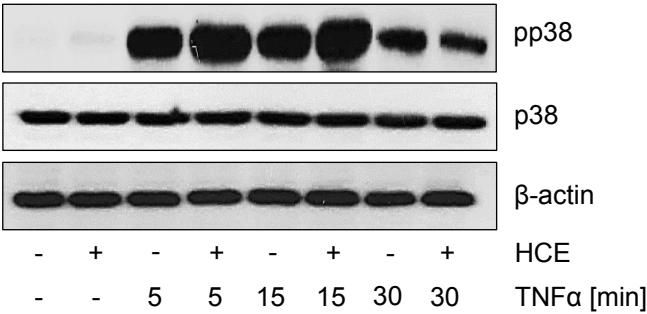

B

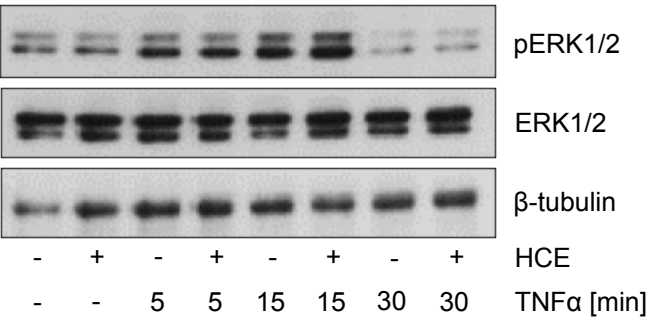

C

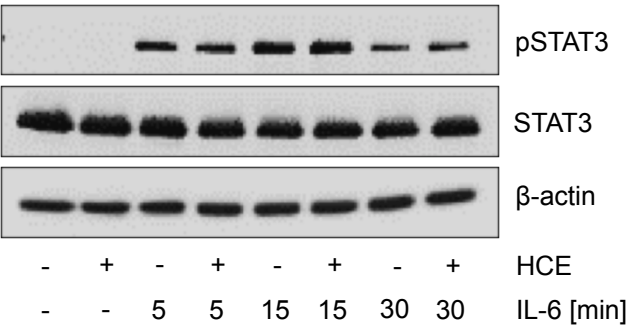

Supplement: Supplementary file 1 [file jcmm0019-1021-sd1.pdf]
