# Supplementary material for: Haemanthus coccineus extract and its main bioactive component narciclasine display profound anti-inflammatory activities in vitro and in vivo
Source: J Cell Mol Med. 2015 Mar 5;19(5):1021–32. doi: 10.1111/jcmm.12493 (PMC4420604; doi:10.1111/jcmm.12493)
Supplement: Supplementary file 2 [file jcmm0019-1021-sd2.docx]

**Supporting information**

**Table 1**: Minimum inhibitory concentration (MIC) of HCE and narciclasine against relevant human pathogens

|  | MIC | | | | |
| --- | --- | --- | --- | --- | --- |
|  | ATCC 29213  *Staphylococcus aureus* | ATCC 29212  *Enterococcus faecalis* | ATCC 25922  *Escherichia coli* | ATCC 27853  *Pseudomonas aeruginosa* | ATCC 9341  *Micrococcus luteus* |
| HCE | > 1 µg/ml | > 1 µg/ml | > 1 µg/ml | > 1 µg/ml | > 1 µg/ml |
| Narciclasine | > 300 nM | > 300 nM | > 300 nM | > 300 nM | > 300 nM |
