# Supplementary material for: Haemanthus coccineus extract and its main bioactive component narciclasine display profound anti-inflammatory activities in vitro and in vivo
Source: J Cell Mol Med. 2015 Mar 5;19(5):1021–32. doi: 10.1111/jcmm.12493 (PMC4420604; doi:10.1111/jcmm.12493)
Supplement: Supplementary file 3 [file jcmm0019-1021-sd3.docx]

**Supporting figure legend**

**Fig. S1** Cell viability is affected by HCE only at very high concentrations. (**A**) ECs were pretreated at various concentrations (10 ng/ml - 10 µg/ml) of HCE for 24 h and during the last 4 h resazurin was added. Fluorescence intensity of the resazurin metabolite resorufin was measured (ex: 560 nm; em: 590 nm). N = 3; **P* < 0.001 versus control. (**B**) Confluent HUVECs were either pretreated for 24 h with HCE (10 ng/ml - 10 µg/ml) or left untreated. Subdiploid DNA content was determined by flow cytometry. N = 3; **P* < 0.001 versus control.

**Fig. S2** HCE does not affect thrombin receptor-activating peptide (TRAP)-induced endothelial hyperpermeability. HMECs were pretreated for 30 min with HCE prior to TRAP (50 µM, 30 min). Control cells were treated with DMSO as vehicle control. The flux of FITC-labelled dextran (1 mg/ml) across endothelial monolayer was measured with a Transwell^®^ two-compartment system. Samples were taken from the lower compartment. N = 3; n.s. = not significantly different.

**Fig. S3** HCE does not interfere with endothelial MAPK or STAT3 signalling pathway. HUVECs were pretreated with 300 ng/ml HCE before incubation with 10 ng/ml TNFα (A, B) or with 10 ng/ml IL-6 (C). Phosphorylation (activation) of p38 MAPK, ERK1/2, and STAT3 were analysed by Western blotting. Tubulin and actin served as loading control. One representative out of 3 independently performed experiments is shown, each.
